# Supplementary material for: Revisiting the hypothesis of an energetic barrier to genome complexity between eukaryotes and prokaryotes
Source: R Soc Open Sci. 2020 Feb 12;7(2):191859. doi: 10.1098/rsos.191859 (PMC7062059; doi:10.1098/rsos.191859)
Supplement: Dataset S1 [file rsos191859supp1.zip › datasetS1/dataset_references.pdf]

# References


Armbrust, E. V. et al. (2004) The genome of the diatom Thalassiosira pseudonana: ecology, evolution, and metabolism. *Science (New York, N.Y.)*. [Online] 306 (5693), 79–86. [online]. Available from: http://www.ncbi.nlm.nih.gov/pubmed/15459382.

Bayen, M. & Dalmon, J. (1975) [Physico-chemical determination of the ploidy of the unicellular alga, Chlorella pyrenoidosa (strain 211/8b) (author's transl)]. *Biochimica et biophysica acta*. [Online] 395 (2), 213–219. [online]. Available from: http://www.ncbi.nlm.nih.gov/pubmed/1138941.

Blake, D. P. et al. (2015) Population, genetic, and antigenic diversity of the apicomplexan Eimeria tenella and their relevance to vaccine development. *Proceedings of the National Academy of Sciences of the United States of America*. [Online] 112 (38), E5343-50. [online]. Available from: http://www.ncbi.nlm.nih.gov/pubmed/26354122.

Blanc, G. et al. (2010) The Chlorella variabilis NC64A Genome Reveals Adaptation to Photosymbiosis, Coevolution with Viruses, and Cryptic Sex. *The Plant Cell*. [Online] 22 (9), 2943–2955. [online]. Available from: http://www.plantcell.org/lookup/doi/10.1105/tpc.110.076406.

Breuert, S. et al. (2006) Regulated polyploidy in halophilic archaea. *PLoS ONE*. [Online] 1 (1), .

Crary, M. (2012) *Genetic Variability and its Relationship to Acanthamoeba Pathogenesis*. Ohio State University. [online]. Available from: http://rave.ohiolink.edu/etdc/view?acc_num=osu1343831120.

Daniels, J.-P. et al. (2010) Cell biology of the trypanosome genome. *Microbiology and molecular biology reviews : MMBR*. [Online] 74 (4), 552–569. [online]. Available from: http://www.ncbi.nlm.nih.gov/pubmed/21119017.

von Dassow, P. et al. (2008) INTER- AND INTRASPECIFIC RELATIONSHIPS BETWEEN NUCLEAR DNA CONTENT AND CELL SIZE IN SELECTED MEMBERS OF THE CENTRIC DIATOM GENUS THALASSIOSIRA (BACILLARIOPHYCEAE). *Journal of Phycology*. [Online] 44 (2), 335–349. [online]. Available from: http://doi.wiley.com/10.1111/j.1529-8817.2008.00476.x.

Drmota, T. & Král, J. (1997) Karyotype of Trichomonas vaginalis. *European Journal of Protistology*. [Online] 33 (2), 131–135. [online]. Available from: https://linkinghub.elsevier.com/retrieve/pii/S0932473997800302.

Dubessay, P. et al. (2001) Effect of large targeted deletions on the mitotic stability of an extra chromosome mediating drug resistance in Leishmania. *Nucleic acids research*. [Online] 29 (15), 3231–3240. [online]. Available from: http://www.ncbi.nlm.nih.gov/pubmed/11470881.

Fenchel, T. & Finlay, B. J. (1983) Respiration rates in heterotrophic, free-living protozoa. *Microbial Ecology*. [Online] 9 (2), 99–122. [online]. Available from: http://link.springer.com/10.1007/BF02015125.

Fokin, S. I. et al. (2001) Nuclear reorganization variety in Paramecium (Ciliophora: Peniculida) and its possible evolution. *Acta Protozoologica*. 40 (4), 249–261.

Gerald, N. et al. (2011) Mitosis in the human malaria parasite Plasmodium falciparum. *Eukaryotic cell*. [Online] 10 (4), 474–482. [online]. Available from: http://www.ncbi.nlm.nih.gov/pubmed/21317311.

Gibson, W. C. & Miles, M. A. (1986) The karyotype and ploidy of Trypanosoma cruzi. *The EMBO journal*. [Online] 5 (6), 1299–1305. [online]. Available from: http://www.ncbi.nlm.nih.gov/pubmed/3525150.

Gorovsky, M. A. (1973) Macro- and micronuclei of Tetrahymena pyriformis: a model system for studying the structure and function of eukaryotic nuclei. *The Journal of protozoology*. [Online] 20 (1), 19–25. [online]. Available from: http://www.ncbi.nlm.nih.gov/pubmed/4632259.

Green, J. C. et al. (1996) The life-cycle of Emiliania huxleyi: A brief review and a study of relative ploidy levels analysed by flow cytometry. *Journal of Marine Systems*. [Online] 9 (1–2), 33–44. [online]. Available from: https://linkinghub.elsevier.com/retrieve/pii/0924796396000140.

Hildenbrand, C. et al. (2011) Genome copy numbers and gene conversion in methanogenic archaea. *Journal of Bacteriology*. [Online] 193 (3), 734–743.

Joo, S. et al. (2017) Gene Regulatory Networks for the Haploid-to-Diploid Transition of Chlamydomonas reinhardtii. *Plant physiology*. [Online] 175 (1), 314–332. [online]. Available from: http://www.ncbi.nlm.nih.gov/pubmed/28710131.

Katz, E. R. & Sussman, M. (1972) Parasexual recombination in Dictyostelium discoideum: selection of stable diploid heterozygotes and stable haploid segregants

(clones-temperature sensitive-ploidy-fruiting bodies-spore-slime mold). *Proceedings of the National Academy of Sciences of the United States of America*. [Online] 69 (2), 495–498. [online]. Available from: http://www.ncbi.nlm.nih.gov/pubmed/4501129.

Makarieva, A. M. et al. (2005) Energetics of the smallest: do bacteria breathe at the same rate as whales? *Proceedings of the Royal Society B: Biological Sciences*. [Online] 272 (1577), 2219–2224. [online]. Available from: http://rspb.royalsocietypublishing.org/cgi/doi/10.1098/rspb.2005.3225.

Makarieva, A. M. et al. (2008) Mean mass-specific metabolic rates are strikingly similar across life's major domains: Evidence for life's metabolic optimum. *Proceedings of the National Academy of Sciences of the United States of America*. [Online] 105 (44), 16994–16999. [online]. Available from: http://www.pubmedcentral.nih.gov/articlerender.fcgi?artid=2572558&tool=pmcentrez&rendertype=abstract.

Maldonado, R. et al. (1994) Changes of ploidy during the Azotobacter vinelandii growth cycle. *Journal of Bacteriology*. [Online] 176 (13), 3911–3919.

Michelsen, O. et al. (2010) The MG1363 and IL1403 laboratory strains of Lactococcus lactis and several dairy strains are diploid. *Journal of Bacteriology*. [Online] 192 (4), 1058–1065.

Mukherjee, C. et al. (2008) Entamoeba shows reversible variation in ploidy under different growth conditions and between life cycle phases. *PLoS neglected tropical diseases*. [Online] 2 (8), e281. [online]. Available from: http://www.ncbi.nlm.nih.gov/pubmed/18714361.

Pecoraro, V. et al. (2011) Quantification of Ploidy in Proteobacteria Revealed the Existence of Monoploid, (Mero-)Oligoploid and Polyploid Species Syed Aziz (ed.). *PLoS ONE*. [Online] 6 (1), e16392. [online]. Available from: https://dx.plos.org/10.1371/journal.pone.0016392.

Reis-Cunha, J. L. et al. (2015) Chromosomal copy number variation reveals differential levels of genomic plasticity in distinct Trypanosoma cruzi strains. *BMC genomics*. [Online] 16499. [online]. Available from: http://www.ncbi.nlm.nih.gov/pubmed/26141959.

De Riso, V. et al. (2009) Gene silencing in the marine diatom Phaeodactylum tricornutum. *Nucleic acids research*. [Online] 37 (14), e96. [online]. Available

from: http://www.ncbi.nlm.nih.gov/pubmed/19487243.

Rollinson, D. (1977) *Biochemical and biological characters of Eimeria species*. University of London. [online]. Available from: https://spiral.imperial.ac.uk/bitstream/10044/1/22766/2/Rollinson-D-1977-PhD-Thesis.pdf.

Romano, A. et al. (2014) Cross-species genetic exchange between visceral and cutaneous strains of Leishmania in the sand fly vector. *Proceedings of the National Academy of Sciences of the United States of America*. [Online] 111 (47), 16808–16813. [online]. Available from: http://www.ncbi.nlm.nih.gov/pubmed/25385616.

Shoguchi, E. et al. (2013) Draft assembly of the Symbiodinium minutum nuclear genome reveals dinoflagellate gene structure. *Current biology : CB*. [Online] 23 (15), 1399–1408. [online]. Available from: http://www.ncbi.nlm.nih.gov/pubmed/23850284.

Slabodnick, M. M. et al. (2017) The Macronuclear Genome of Stentor coeruleus Reveals Tiny Introns in a Giant Cell. *Current Biology*. [Online] 27 (4), 569–575. [online]. Available from: https://linkinghub.elsevier.com/retrieve/pii/S0960982216315391.

Tobiason, D. M. & Seifert, H. S. (2006) The Obligate Human Pathogen, Neisseria gonorrhoeae, Is Polyploid Jonathon Eisen (ed.). *PLoS Biology*. [Online] 4 (6), e185. [online]. Available from: https://dx.plos.org/10.1371/journal.pbio.0040185.

Willhoeft, U. & Tannich, E. (1999) The electrophoretic karyotype of Entamoeba histolytica. *Molecular and biochemical parasitology*. 99 (1), 41–53. [online]. Available from: http://www.ncbi.nlm.nih.gov/pubmed/10215023.

Xu, W. D. et al. (1998) Chromosome numbers of Tritrichomonas foetus and Tritrichomonas suis. *Veterinary parasitology*. 78 (4), 247–251. [online]. Available from: http://www.ncbi.nlm.nih.gov/pubmed/9786624.

Yang, Q. et al. (1994) Sequence organization of the Acanthamoeba rRNA intergenic spacer: identification of transcriptional enhancers. *Nucleic acids research*. [Online] 22 (22), 4798–4805. [online]. Available from: http://www.ncbi.nlm.nih.gov/pubmed/7984432.

Yuh, Y. S. et al. (1997) Chromosome number of Trichomonas vaginalis. *The Journal of parasitology*. [Online] 83 (3), 551–553. [online]. Available from: http://www.ncbi.nlm.nih.gov/pubmed/9194850.
