# Supplementary material for: Revisiting the hypothesis of an energetic barrier to genome complexity between eukaryotes and prokaryotes
Source: R Soc Open Sci. 2020 Feb 12;7(2):191859. doi: 10.1098/rsos.191859 (PMC7062059; doi:10.1098/rsos.191859)
Supplement: Dataset S1 [file rsos191859supp1.zip › datasetS1/README.pdf]

Dataset S1. Datasets used in this study.

The files “data_prokaryotes.csv” and “data_eukaryotes.csv” are the original datasets of prokaryotes and eukaryotes used in this study, respectively. Each table includes the species name, ploidy level range (columns “Ploidy_level_min” and “Ploidy_level_max”), genome size (bp; column “Nuc”), gene number (column “Gene”), and species identifier (column “NAME”) that corresponds to the Kyoto Encyclopedia of Genes and Genomes (KEGG) organism ID (see www.genome.jp/kegg/catalog/org_list.html), mass-specific metabolic rate (Wkg^{-1}; column “qWkg_W_kg”), cell mass (pg; column “Mpg_pg”), and temperature (C^{o}; column “Temp”). The columns “Nuc_Ref” and “Gene_Num_Ref” denote the references for genome size and gene number, respectively. These references are described as KEGG organisms ID or National Center for Biotechnology Information (NCBI) Assembly ID. When the reference is described as “KEGG: xxx”, genome size and/or gene numbers are obtainable from https://www.kegg.jp/kegg-bin/show_organism?org=xxx. When the reference is described as “NCBI: xxx”, genome size and/or gene numbers are available from https://www.ncbi.nlm.nih.gov/assembly/xxx/. The columns “Ploidy_level_Ref”, “qWkg_Ref”, “Mpg_Ref”, and “Temp_Ref” correspond to the references to ploidy level mass-specific metabolic rate, cell mass, and temperature, respectively. See the file “dataset_references.pdf” for these references. The columns with suffix of “Note” denote explanatory remarks; for example, the column “Nuc_Ref_Note” denotes the explanatory remark of the genome size. The columns beginning with “qWkg_Note_” denote the comments on metabolic rate measurements. Their references correspond to the column “qWkg_Ref”.

The file “summary_metabolic_rate_types.csv” is the summary of metabolic rate types. The table includes a species category [i.e., eukaryote (eu) or prokaryote (pro)], species name, and species identifier (column “id”) that correspond to the Kyoto Encyclopedia of Genes and Genomes (KEGG) organism ID (see www.genome.jp/kegg/catalog/org_list.html), metabolic rate type, comments on metabolic rate measurements (columns starting with “comment”), mass-specific metabolic rate (Wkg^{-1}; column “qWkg_W_kg”) and cell mass (pg; column “Mpg_pg”). The metabolic rate types were defined based on the comments on metabolic rate measurements and references: metabolic rate measured at the death phase (Death), endogenous metabolic rate (Endogenous), metabolic rate of growing cell (Growth), metabolic rate measured at the exponential or logarithmic growth phase (Log), metabolic rate of starved cells (Starved), metabolic rate measured at the stationary growth phase (Steady), summit metabolic rate (Summit), and metabolic rate measurements unclearly explained (Unknown). The references for the comments on metabolic rate measurements correspond to the column “qWkg_Ref”.

The file “standard_statistical_analysis.R” is an R source code for generating the dataset used in the standard statistical analyses and for performing the standard statistical analyses (the statistical tests and standard logistic regression analyses).

The file “phylogenetic_analysis.R” is an R source code for generating the dataset used in the phylogenetic logistic analyses and for performing phylogenetic logistic regression

analyses.

The “ML_phylogenetic_tree_LG_G.nwk” is the phylogenetic tree presented in the Newick format. The tree is used in the phylogenetic logistic analyses (see also electronic supplementary material, figure S1).
