# Supplementary material for: Revisiting the hypothesis of an energetic barrier to genome complexity between eukaryotes and prokaryotes
Source: R Soc Open Sci. 2020 Feb 12;7(2):191859. doi: 10.1098/rsos.191859 (PMC7062059; doi:10.1098/rsos.191859)
Supplement: Figure S1 [file rsos191859supp2.docx]

**Figure S1. Phylogenetic tree used for phylogenetic logistic regression analysis.**

The node labels correspond to the Kyoto Encyclopaedia of Genes and Genomes (KEGG) organisms ID (see www.genome.jp/kegg/catalog/org_list.html). See the tree presented in the Newick format (ML_phylogenetic_tree_LG_G.nwk in electronic supplementary material, dataset S1).
