# Supplementary material for: Revisiting the hypothesis of an energetic barrier to genome complexity between eukaryotes and prokaryotes
Source: R Soc Open Sci. 2020 Feb 12;7(2):191859. doi: 10.1098/rsos.191859 (PMC7062059; doi:10.1098/rsos.191859)
Supplement: Table S2 [file rsos191859supp4.docx]

**Table S2. Contributions of explanatory variables to the prokaryote–eukaryote dichotomy when removing prokaryotes whose ploidy level has not been reported in any previous studies.** In total, 35 prokaryotes and 36 eukaryotes were considered for these data analyses. The variable “Dichotomy” indicates whether a species is a eukaryote (1) or not (0). SE and AIC correspond to the standard error and Akaike information criterion value of the model, respectively. *R*^2^ is the coefficient of determination.

| Model | Variable | Estimate | SE | *p*-value | AIC | *R*^2^ |
| --- | --- | --- | --- | --- | --- | --- |
| (a) Dichotomy ~ Power per genome + Cell mass | Power per genome | 3.15 | 1.93 | 0.10 | 16.4 | 0.90 |
|  | Cell mass | 9.21 | 5.51 | 0.095 |  |  |
| (b) Dichotomy ~ Power per gene + Cell mass | Power per gene | ­2.10 | 1.40 | 0.13 | 17.9 | 0.89 |
|  | Cell mass | 10.23 | 5.23 | 0.050 |  |  |
| (c) Dichotomy ~ Power per genome | Power per genome | 6.52 | 1.79 | 2.6×10^–4^ | 25.2 | 0.80 |
| (d) Dichotomy ~ Power per gene | Power per gene | 4.61 | 1.17 | 7.7×10^–5^ | 40.0 | 0.68 |
| (e) Dichotomy ~ Cell mass | Cell mass | 11.08 | 4.30 | 0.0099 | 19.3 | 0.85 |
